# The Impact of Wood Waste on the Properties of Silicone-Based Composites

**DOI:** 10.3390/polym13010007

**Published:** 2020-12-22

**Authors:** Maciej Mrówka, Małgorzata Szymiczek, Magdalena Skonieczna

**Affiliations:** 1 Department of Theoretical and Applied Mechanics, Silesian University of Technology, Konarskiego 18 A, 44-100 Gliwice, Poland; Maciej.Mrowka@polsl.pl; 2 Department of Systems Biology and Engineering, Silesian University of Technology, Akademicka 16, 44-100 Gliwice, Poland; Magdalena.Skonieczna@polsl.pl; 3 Biotechnology Centre, Silesian University of Technology, Krzywoustego 8, 44-100 Gliwice, Poland

**Keywords:** wood–plastic composite (WPC), silicone, mechanical properties, cytotoxicity, casting, ageing

## Abstract

The impact of wood waste on the mechanical and biological properties of silicone-based composites was investigated using wood waste from oak, hornbeam, beech, and spruce trees. The density, abrasion resistance, resilience, hardness, and static tensile properties of the obtained WPC (wood–plastic composites) were tested. The results revealed slight changes in the density, increased abrasion resistance, decreased resilience, increased hardness, and decreased strain at break and stress at break compared with untreated silicone. The samples also showed no cytotoxicity to normal human dermal fibroblast, NHDF. The possibility of using prepared composites as materials to create structures on the seabed was also investigated by placing samples in a marine aquarium for one week and then observing sea algae growth.

## 1. Introduction

Polymeric fillers are very popular and include natural fillers, which can be derived from both vegetables (maize husk, nutshell, ground coffee, bran, and starch) and animals (milled bird feathers). Such fillers are introduced primarily into thermoplastic polymer materials [1,2,3,4]. An important feature of this type of material, apart from the ability to change their physical and mechanical properties, is the ability to modify their environmental properties. Wood–plastic composite (WPC) with a thermoplastic matrix, which are mainly obtained by extrusion and injection, have found applications in the construction industry (boards, furniture, and lagging) [2,4,5,6]. The introduction of biofillers for duroplasts, along with changes in the physical and mechanical properties, allows resin shrinkage to be minimized, but it also increases the resin viscosity, making it difficult to saturate fabrics. A separate issue is the introduction of silicone biofillers, which are used for both sealing and construction elements [2,5,6]. The main problem in obtaining polymer–wood composites is the lack of compatibility between the hydrophilic wood waste filler and hydrophobic polymer matrices. To ensure proper adhesion between the filler and matrix, it is important to treat the surface of wood waste to make it hydrophilic, for example by using acetic anhydride and thermal treatment [2,6,7,8,9]. Thermal treatment of wood waste causes changes in color, regrouping of polymers, and in the case of resinous trees, modification and redistribution of wood extracts [9,10]. The result is increased measurement stability and resistance to biodegradation, and lower mechanical properties. Previous research has shown that increasing the strength and stiffness is affected by the shape of the waste and not its size [11]. Wood–polymer composites may also prove to be alternatives to traditional materials used for facade panels, concrete fillers, packaging, and protective and anticorrosion coatings. These materials are especially interesting due to environmental concerns [1,2,6,12]. Silicone-based composites are one of the most important elastic technical materials produced industrially, e.g., glass-fiber-reinforced silicone composites [13]. In a high-power white light emitting diode (LED) package, the phosphor-silicone composite is typically used for photometric and colorimetric conversions, ultimately producing the white light [14]. Ceramifiable silicone rubber composites play important roles in the field of thermal protection systems (TPS) for rocket motor cases due to their advantages [15]. There are ceramizable (ceramifiable) silicone-based composites commonly used to increase flame retardancy of electrical cables and to ensure integrity of electricity network during fire by their ability to create a continuous ceramic structure [16]. In other paper silicone composites filled with different-sized nickel particles. The samples with particles showed larger improvements in shear storage modulus than those without particles [17]. By using an appropriate filler, composites with the desired properties can be obtained. The silicone-based membrane with 0.36 wt % of graphene oxide showed excellent antifouling performance, and is promising in practical applications [18]. There are no articles in the literature devoted to the introduction of fillers of natural origin into silicones. The authors took up this topic due to the need to create new materials based on waste products of natural origin.

This work aimed to assess the impact of wood waste from deciduous (beech, oak, and hornbeam) and coniferous trees (spruce) on the mechanical and biological properties of silicone composites used for structural element protection. Such a goal required the use of several physical and mechanical tests (static tensile test, hardness, abrasiveness, and density) and biological tests, which included cytotoxicity and aging tests in a replicated seawater environment.

## 2. Materials and Methods

### 2.1. Materials

The research was carried out on silicon matrix composites XIAMETER 4234-T4 (additive silicone) from Dow Corning (Table 1), in which the filler was wood waste from deciduous trees: oak (*Quercus robur*), beech (*Fagus sylvatica*), and hornbeam (*Carpinusbetulus*), and coniferous spruce (*Piceaabies*). Tree properties are summarized in Table 2. To identify the fillers, microscopic photos were taken on a stereo discovery Zeiss stereoscopic microscope (Carl Zeiss AG, Oberkochen, Germany), and single particles were imaged on a Zeiss Supra 35 scanning electron microscope, (Carl Zeiss AG, Oberkochen, Germany). The particle size distribution was tested on the Fritsch Analysette 22 Micro Tec Plus (FRITSCH GmbH, Idar-Oberstein, Germany). 

### 2.2. Methodology

#### 2.2.1. Composites

The composites were prepared by gravity casting with a content of 10 and 20 wt % wood waste. The mark of the composites is shown in Table 3.

Before introducing wood waste into the silicone matrix, it was gradually heat-treated at 180°C for 180 min, until a constant weight was obtained. The dried waste was sieved to obtain particles smaller than 1 mm. Unification of the size of wood waste helped evenly distribute the filler in the obtained composites. Smaller waste sizes also promoted homogenous functional properties throughout produced composites. The silicone component A was mixed with the fillers on a high shear mixer, then the catalyst was added. The mixing speed was 500 RPM.

**Figure 1:**
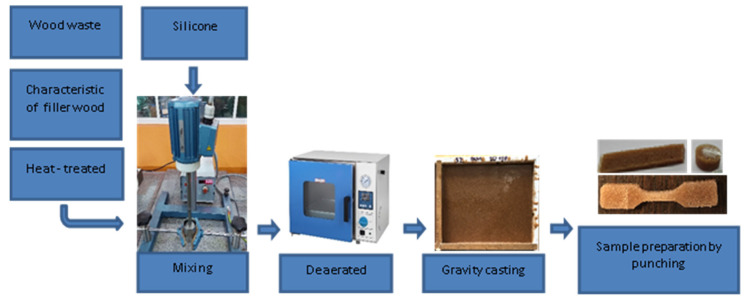
Scheme of preparation samples.

The prepared compositions were deaerated in a vacuum oven for 45 min at 0.78 bar and then cast into previously prepared, leveled molds to ensure a constant plate thickness of 5 mm. Seventy-two hours after being poured into molds, samples were cut by punching. The sample preparation scheme is shown in Figure 1.

The obtained samples were subjected to mechanical and biological tests. Mechanisms were examined using hydrostatic weighing tests, radiation resistance tests, Schopper abrasion resistance tests, and Shore type A hardness tests and static tensile tests. Toxicity to normal human cells was also evaluated using the MTT test. The possibility of using the received WPC to obtain cable covers, which are placed on the seabed was also examined. All tests were carried out at the temperature in the room where the test was performed, which was 22 °C with a humidity of 50%.

#### 2.2.2. Density Testing by Hydrostatic Weighing

Densities were measured on a scale in accordance with EN ISO 1183-1:2006 using 5 samples from each system [24]. The method of determining the density by the method of hydrostatic weighing of composite polymer materials consisted in weighing the test sample with the use of an OHAUS Adventurer-Pro (OHAUS Europe GmbH, Nänikon, Switzerland) analytical balance with a density measurement kit. The sample was weighed twice. The first measurement is carried out with the sample placed on the pan and surrounded by air. The second measurement is carried out for a sample immersed in a liquid of known density. During the tests, water was used as a liquid with a known density *d* = 0.997 g/cm^3^.

Density was determined using Formula (1):

(1) $$d=d_{H20}\frac{m_{1}}{\left(m_{1}-m_{2}\right)}$$

where:

- *d_H_*_20_—density of water (g/cm^3^),
- *m*_1_—dry sample mass (g),
- *m*_2_—wet sample mass (g).

#### 2.2.3. Rebound Resilience with Schober’s Test

The resilience test was carried out in accordance with the EN ISO 4662:2017 standard for five samples with dimensions of 30 mm × 30 mm × 5 mm [25]. Before conducting basic tests, the samples were mechanically conditioned (2 impacts). The measurement of the resilience of composite materials consists in hitting the sample with a weight placed on a pendulum. The sample is held in an anvil attached to a metal body. The measurement consisted in reading the value indicated by the pointer on the value axis (%).

#### 2.2.4. Abrasion Resistance Tests

The abrasion resistance tests were carried out on a Schopper–Schlobach apparatus (APGI) in accordance with the ISO 4649:2007 standard [26]. In the research, sandpaper (60 grit) was used, wound on a roller with a diameter of 150 mm, which was rotating at a speed of 40 RPM. Abrasion resistance was determined for 3 cylindrical samples with a 16 mm diameter and 10 mm height. Due to the thickness of spilled boards (5 mm), the samples were glued with cyanoacrylate adhesive (according to ISO 4649: 2007). Abrasion resistance (abrasive wear), i.e., the volume loss relative to a standard sample, was determined based on the Formula (2):

(2) $$\Delta V=\;\frac{m_{1}-m_{2}}{d}$$

where:

- *m*_1_—mass of sample before abrasion (g),
- *m*_2_—sample mass after abrasion (g),
- *d*—sample density (g/cm^3^).

#### 2.2.5. Hardness Test

The shore A hardness test was carried out in accordance with ISO 7619-1:2010 [27]. The measurements were made with a hardness durometer Shore A type (Etopoo). Five measurements were taken on each of the composites, maintaining a distance of at least 10 mm from the sample edge.

#### 2.2.6. Tensile Test

Tensile strength tests were performed in accordance with EN ISO 527-1 [28]. The measurements were carried out according to EN ISO 527-1 [28] for 5 samples (type 5-B) cut from each composition and native samples. The test was carried out on the Instron 4465 tensile test machine. The test speed was 50 mm/min. The stress at break and strain at break were determined.

#### 2.2.7. Cytotoxicity Testing of Composite Samples

Cytotoxicity testing of composite samples according to the procedure described earlier [29,30]. Cell viability of normal human dermal fibroblasts (NHDF, Lonza) was assessed using an MTT (3-[4-5-dimethylthiazol-2-yl]-2,5-diphenyltetrazolium bromide) test. Cells were seeded in Petri plates with a concentration of 10^5^ cells per well. Cell cultures were seeded on tested materials and incubated for 72 h at 37 °C in a humidified atmosphere with 5% CO_2_. Then, the culture medium was removed and replaced with a trypsin solution for cell collection. After trypsin neutralization, cell suspensions were centrifuged (2000 RPM, 3 min, room temperature) and cell pellets were resuspended in the MTT solution (50 µL, 0.5 mg/mL in RPMI 1640 without phenol red, Sigma, (Saint Louis, MO, USA). After 3 h of incubation, the MTT solution was removed, and the acquired formazan was dissolved in isopropanol:HCl system. Finally, the absorbance at 570 nm was spectrophotometrically measured with a plate reader Epoch, BioTec, (Winooski, VT, USA). The results were expressed as a survival fraction (%) in comparison to the untreated controls (100%), from 3 separate experiments, ±SD.

#### 2.2.8. Ageing in Seawater Conditions

Due to the potential application of the prepared materials for the covers of structures installed in the seabed, tests were carried out in the sea water environment. The possibility of surface settling of obtained composites was investigated using marine algae. The tests were carried out in an aquarium with samples mounted on a wooden plate. Seawater was prepared in accordance with the standard ASTM D 1141-52 [31]. The chemical composition of the substitute seawater is shown in Table 4.

Marine algae was added to the aquarium, which was intensively illuminated for 30 days. After this time, the sample plate was removed. The aging was carried out at the temperature of 30 °C, while illuminating the aquarium with a reflector placed 1 m from the aquarium.

## 3. Results and Discussion

### 3.1. Characteristics of Fillers

**Figure 2:**
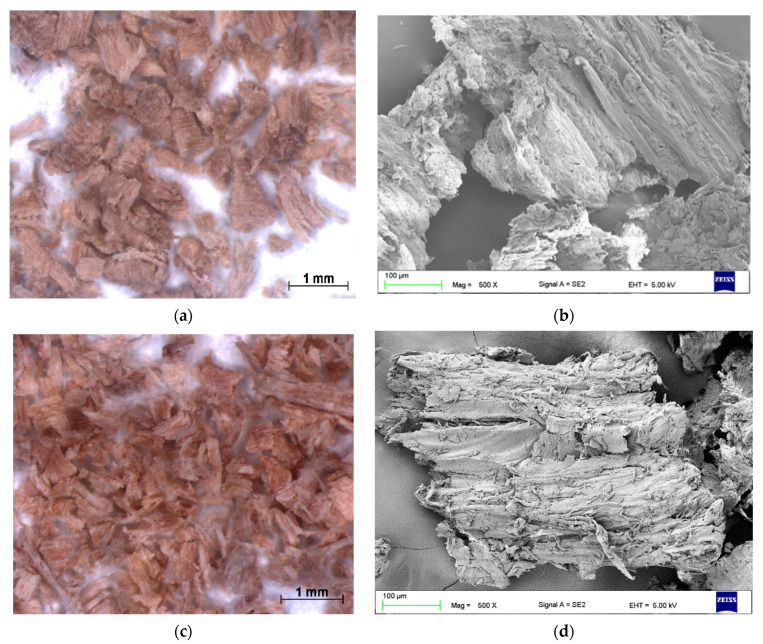

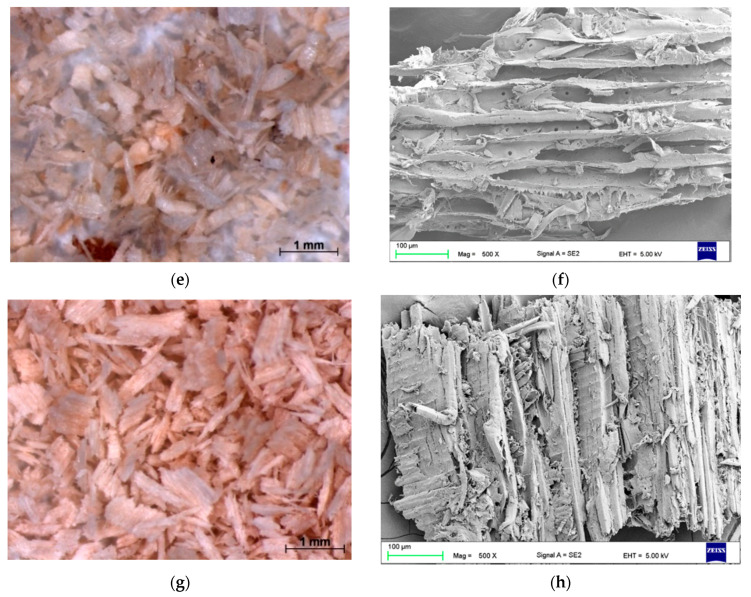
Microscopic image oak (**a**,**b**), beech (**c**,**d**), spruce (**e**,**f**), and hornbeam (**g**,**h**).

**Figure 3:**
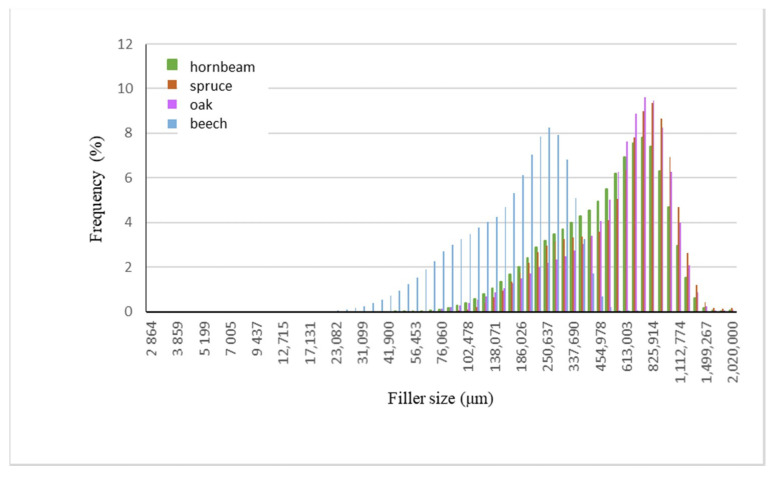
The frequency curve for the tested fillers.

Wood waste fillers are shown in Figure 2. Images of wood fillers were taken on a stereo discovery Zeiss stereoscopic microscope (Figure 2a,c,e,g) and single filler particles were imaged on a Zeiss Supra 35 scanning electron microscope (Figure 2b,d,f,h). Particle size tests were performed on a Fritsch Analysette 22 Micro Tec Plus equipped with a wet dispersing unit. Measuring range 0.08–2000 microns. The particle size distribution is shown in Figure 3. The frequency curves were developed on the basis of 5 measurements for each wood waste. As can be seen, the smallest filler particles were observed for beech and hornbeam.

Analyzing the images of fillers presented in Figure 2, it can be noticed that the structure of spruce differs from that of other trees. Spruce has a tubular structure. The structure of spruce is characterized by visible tracheas and pits, which, for example, increases the mechanical adhesion between the matrix and the filler. However, the release of resin without proper preparation of the spruce filler may adversely affect some properties. Microscopy images of fillers used in this study showed no significant differences in shape, but differed in surface image. These differences can primarily be seen between deciduous tree waste and spruce.

**Figure 4:**
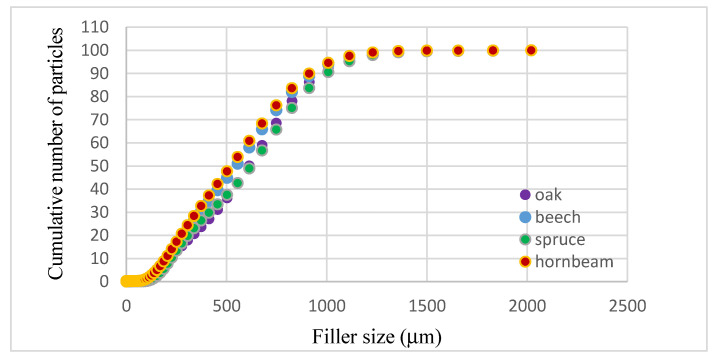
Cumulated curves of the filler’s size.

The size of the most common particles was 747.78 μm for beech (8.2%), hornbeam (7.8%), and oak (9.6%), and 825.9 μm for spruce (9.3%). As can be seen, the smallest particles are characteristic of beech (90%—940.03 μm) and hornbeam (90%—917.03 μm), while the largest range was noted for oak (90%—998.13 μm), while for spruce 90% of the particles have the size below 971.77 μm (Figure 4). This is due to the structure and properties of individual trees.

### 3.2. Density Test Results

**Figure 5:**
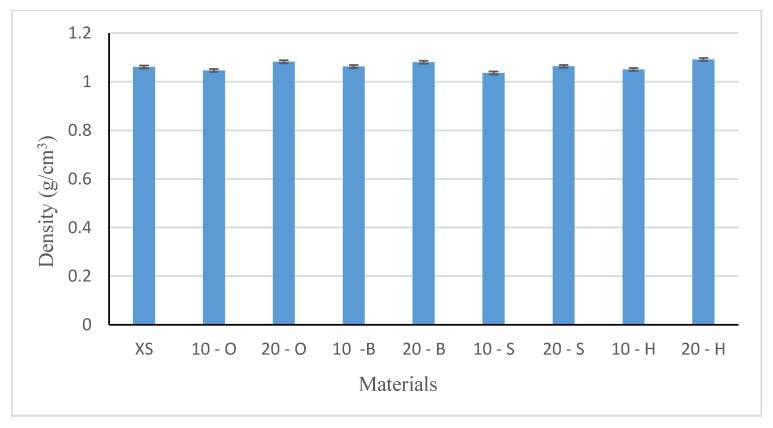
Densities of tested materials (g/cm^3^).

The density of composites produced using wood waste filler were determined by measuring five samples. The average values and their standard deviations are graphically displayed in Figure 5.

Samples from XIAMETER 4234-T4 silicone showed a density of 1.069 g/cm^3^; the specified density was 1.1 g/cm^3^. The introduction of 10% filler, regardless of the type of wood waste, reduced the density of the obtained composites, and the lowest density was obtained using spruce wood waste. The density of the spruce-silicone composite decreased by approximately 5% compared to the native sample (XS). It was most likely caused by the structure of spruce waste as shown in Figure 2f. This is probably the result of tracheas (Figure 2f) distributed throughout the wood, which is why spruce is considered to be a light tree (range 410–500 kg/m^3^, and after drying 700–850 kg/m^3^) and the measurement method. The highest density at 10% filling was recorded for hornbeam, approximately 3% in relation to spruce. This is mainly due to the density and structure of the wood. The hornbeam has a uniform and fine structure, which affects its properties. Hornbeam, beech, and oak belong to heavy and very heavy trees, whose densities after reach 1200 kg/m^3^ (Table 2). Buk contains numerous wood rays, which serve as conductive channels. Oak wood, unlike beech wood, is characterized by wide conductive channels but in much smaller quantities. Composites in which the share of wood waste was 20% had a similar or slightly higher density than native silicone. Composites with 20% filling with hornbeam waste were characterized by the highest density by approximately 4% compared to XS. The conducted one-way analysis of variance showed a significant influence of the examined factors on the density of composites (F = 15.71, test F = 2.208).

### 3.3. Rebound Resilience Results

**Figure 6:**
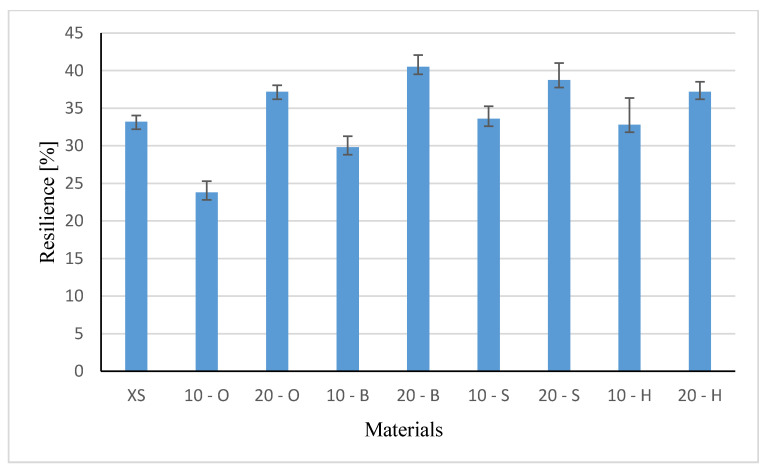
Resilience values of tested materials (%).

The average resilience values and standard deviations of the tested materials are shown in Figure 6.

Introduction 10 wt % wood waste caused a reduction in elasticity for composites with oak by 27% and beech by 20% in relation to native samples, but no change was observed in composites with spruce filling, while a slight increase was observed in the resilience of samples with hornbeam filling. Such a reduction may be related to the size of the grain introduced into the composite and the structure of the wood. The beech particles were characterized by the smallest dimensions, which are confirmed by the normal distribution—Figure 3. Although the literature reports [11] a greater impact on the properties of composites, the shape of the particle has a greater impact, and in the examined case the size is also important. It seems that this is also the result of obtaining the filler from the waste and the properties of the tree itself.

In all types of composites containing 20 wt % filling the wood, resilience increased, the highest of which was observed in samples with beech filling (41%). In composites with hornbeam filling, the resilience results were relatively similar (34.6 and 36.6%). Hornbeam wood is very hard and difficult to break, hence the resilience remained at a similar level, regardless of the content. Regardless of the wood’s cleavage and its hardness, the additional filler improved the flexibility in all systems containing 20 wt % filler. It can therefore be concluded that the introduction of 20 wt % of the wood filler into the systems, regardless of the wood type, its structure, and the particle size, improved the resilience of the obtained composite. That is why the samples recovered their original shape after applying more force than in the unmodified system. The carried out one-way analysis of variance showed that both the content and the type of filler had a significant impact on the change of relativity (F = 38.47, F = 2.208 test).

### 3.4. Abrasion Resistance Results

**Figure 7:**
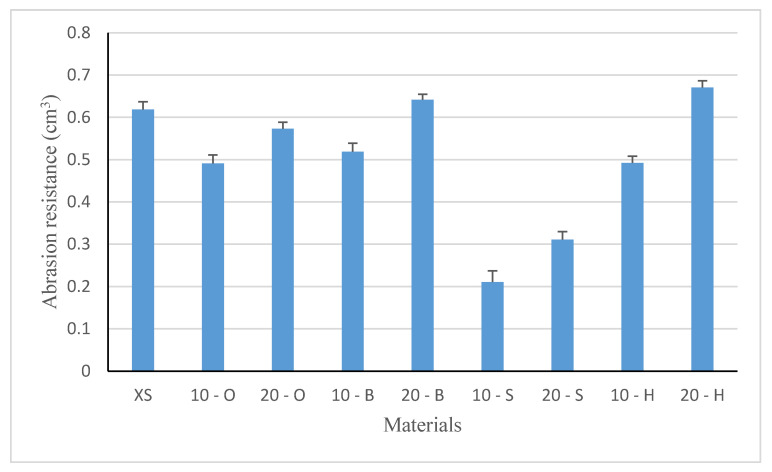
Abrasion resistance of composites (cm^3^).

The results of abrasion resistance tests were compared to a standard sample made of silicone XIAMETER 4234-T4 without filler. The average abrasion resistance values are shown in Figure 7. The test results show that the introduction of filler into the matrix reduced abrasion. The composite containing 10 wt % beech waste (10 - O) showed a loss of 0.51 cm^3^ and had the highest loss of volume. In the case of fillers obtained from deciduous trees, abrasion is approximately 20% lower than that of the native sample. Spruce waste causes a loss of volume by 65% compared to the native sample. The introduction of 20% beech and hornbeam fillers did not affect the abrasion resistance. The differences observed fall within the potions of errors. However, it can be assumed that the distribution of filler particles in the structure of the composite resulted in no changes observed. These fillers are characterized by the smallest particles: beech 90%—940.03 µm and hornbeam 90%—917.03 µm (Figure 4). The lowest abrasion (approximately 50% of XS abrasion) was observed for spruce waste, similarly to composite materials with 10% filling. It seems that the related abrasion is determined mainly by the type of wood and particle size. The smaller the particle, the greater the loss in volume. Samples filled with deciduous tree waste are characterized by worse abrasion resistance than composites with spruce waste. The conducted one-way analysis of variance confirms the strong relevance of the studied factors for the abrasion of composites (F = 202.9, test F = 2.51).

### 3.5. Shore A Hardness Test

**Figure 8:**
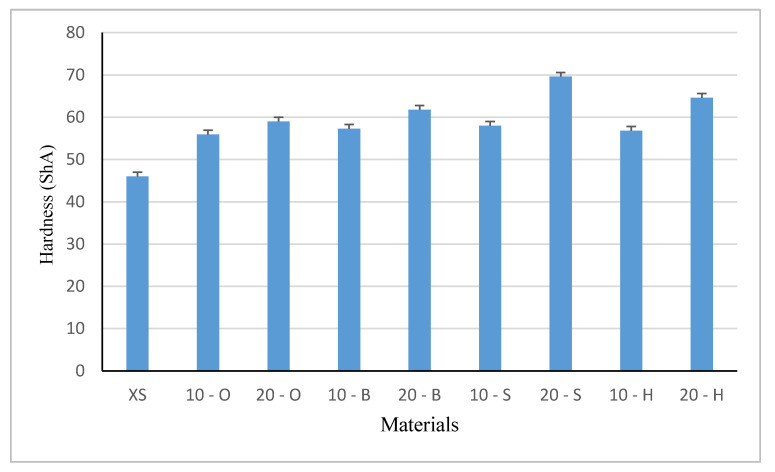
Hardness value of samples (ShA).

Five samples of each composite variant were tested for hardness tests. Graphical presentations of hardness test results and standard error are presented in Figure 8.

In each modified system, the obtained hardness was higher than the unmodified sample. In systems containing 10 wt % XIAMETER 4234-T4 silicone, the obtained composites had comparable hardness values. Both heavy woods like beech, hornbeam, oak, and light spruce showed a hardness of around 57 ShA. Such a high value in the case of spruce precipitation may indicate the content of the resin inside the waste. As in previous systems, when 20 wt % filler was used, the highest value was obtained in samples containing spruce waste. The obtained value of 69.6 ShA was 11 ShA higher than hard oak waste samples.

This may prove that the separated spruce wood resin additionally increased the hardness of the composite, which was also confirmed by the results of resilience. Relatively soft spruce wood did not differ from harder beech, hump, or oak, even taking into account the particle size. As can be seen, as in previous studies, both the type of wood waste and the size of the particle had a significant impact on the obtained hardness results (F = 91.1, test F = 2.208).

### 3.6. Tensile Test Results

**Figure 9:**
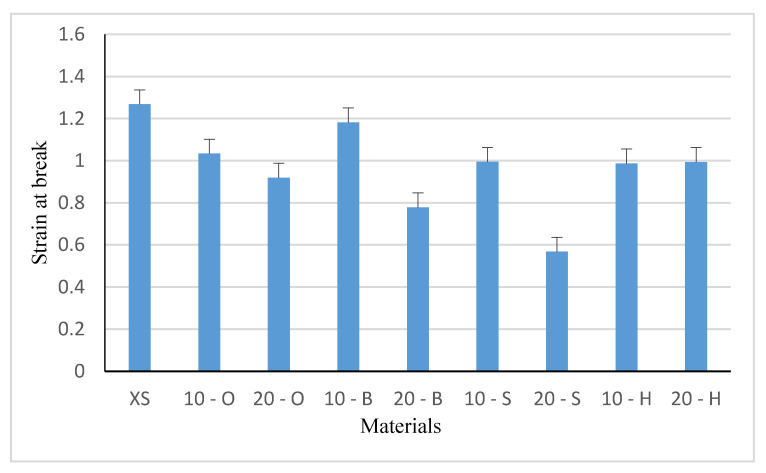
Strain at break.

**Figure 10:**
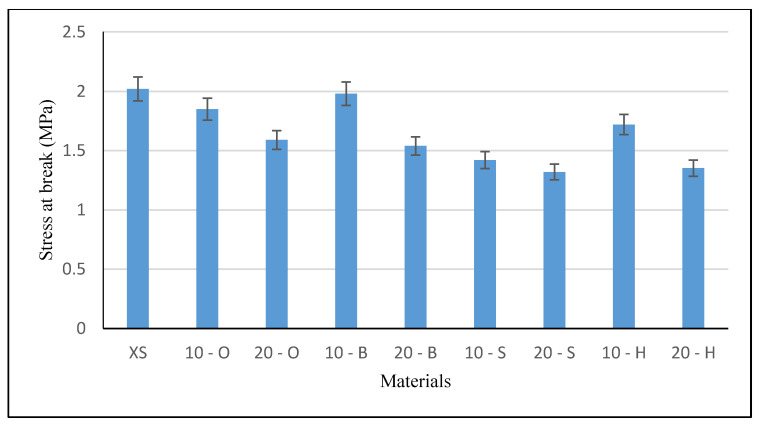
Stress at break (MPa).

Tensile test samples were cut using a punch, which ensured a repeatable shape and dimensions of tested samples. Five measurements were made for each composite variant (filler fraction, type of wood waste). A graphical presentation of the results is shown in Figure 9 (strain at break) and Figure 10 (stress at break).

Samples of pure XIAMETER 4234-T4 silicone showed the greatest strain at break, and all other composites showed reduced strain at break. The sample showing the most similar strain to unmodified silicone (1.269) was that with 10 wt % beech content (1.181). The lowest value was observed in composite 20 - S (0.567), but also the highest measurement error of 6% and standard deviation of 0.06, which may be a result of the preparation of the filler for introduction, the introduction technology itself or the physicochemical properties of wood. Composites containing hornbeam filling showed similar deformation values, irrespective of the percentage of the composite (0.994 and 0.985, respectively). This seems to be related primarily to the distribution of the filler in the composite, and secondly to the grain size. The one-way analysis of variance showed a significant impact on the stain values of the type and content of the filler (F = 162.19, test F = 2.208).

It can be observed that the smaller the filler particles and the proportion by weight, the better the strain at break, which confirms the adopted theories. The lowest strain at break was observed for composites with 20% spruce filling, which proves a significant influence of wood properties on the tested composite characteristics.

The tensile test results indicate that a higher stress at break was observed in samples modified with XIAMETER 4234-T4 silicone. All composites showed lower stress at break, with the exception of composite 10 - B, whose value (1.98 MPa) was similar to unmodified silicone (2.02 MPa). The greatest differences between the native and filled samples were observed for composites with meringue waste and amounted to 10 wt %—30% and for 20 wt %—35%. The composites with the filler from deciduous trees had smaller differences. For 10%, the filling was on average approximately 8% (the lowest value was shown by hornbeam—1.72 MPa), while for 20% by wt filling 26%. ANOVA analysis confirmed the significant influence of the tested factors on the stress at break, F = 35.75, test F = 2.208.

### 3.7. Cytotoxicity Test Results

**Figure 11:**
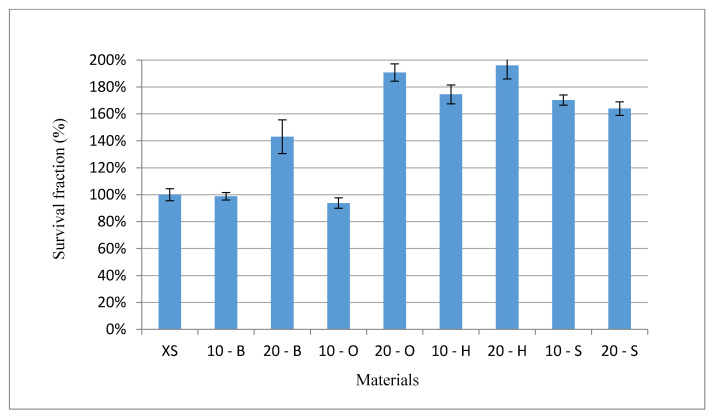
Cytotoxicity test results (%).

The cytotoxicity of the tested composites was obtained according to the standard procedures against model cell lines [29,30,32]. The viability and proliferation assays, such as an MTT assay, allowed for biocompatibility of different, natural, or synthetic agents assessments [32,33]. Figure 11 presents the cytotoxicity against NHDF cells. The cytotoxicity test results showed that neither silicon nor any of the tested composites showed any toxicity to the normal fibroblasts. After 72 h, the fraction of living cells for the XS, 10 - B, and 10 - O groups showed similar values of 100%, 98%, and 93%, respectively. The remaining results showed significant cell proliferation when in contact with composites. The 20 - O and 20 - H composites showed the most proliferative values, for which the living cell fractions were 190% and 196%, respectively. The results confirm the biocompatibility of composites and any toxicity against the NHDF cell line in a standard in vitro cytotoxicity assay [34] was reported.

### 3.8. Possibly of Using WPC on the Seabed

**Figure 12:**
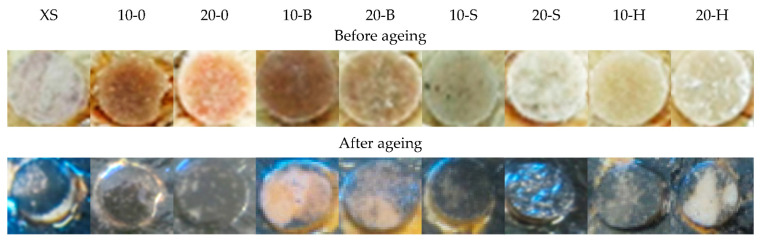
Comparison of plates with composites before and after immersion in a marine aquarium.

Figure 12 shows the image of composite samples before and after ageing in seawater.

Based on the observation of plates covered with marine algae, it can be concluded that algae covered the composites with a higher wood waste content. The exception is the composite containing hornbeam waste in which higher algae content was observed on the sample containing 10 wt % wood waste. The silicone sample was also overgrown by marine algae. Most importantly, in all the samples tested, there is a larger or smaller sample of marine algae. After performing additional tests, this will enable the use of the tested composites as materials for use on the seabed. The results of this research are presented in Table 5.

Microscopy images of samples aged in seawater showed the growth of marine algae film on their surface. Along with filler content, the number of marine algae increased and the color of the silicone matrix changed. At the same time, it should be noted that in the case of spruce waste, a smaller algae growth rate was observed compared with composites filled with deciduous tree waste. Samples with 10 wt % filler showed more diffuse growth than those with 20 wt % filler. Composites containing hornbeam waste were the most overgrown, while the least overgrown were those with beech filling, possibly due to the hardness of the tree (beech shows the lowest hardness among deciduous trees). This also explains the observations of the spruce composite filling. It can therefore be concluded that more algae growth occurred on harder trees (i.e., those containing more cellulose fibers).

In order to select the best filler and content, a simplified multicriteria assessment table was developed (Table 6). The grading scale was adopted from 1 to 5, where 5 means the composites with the best properties and 1 the worst. The same grade was assigned for identical averaged test results. Assessments were made for each percentage of filling separately, based on the results of the tests carried out. The results of the cytotoxicity and ageing tests were not included in the multicriteria analysis. Cytotoxicity measured in the MTT test showed no cytotoxicity of all materials against normal cells, NHDF and more accurate interaction of materials with cells requires additional tests. In turn, the aging was assessed on the basis of macro and microscopic photos of the surface, which makes it impossible at this stage to assess changes in the mechanical and physicochemical properties of the materials.

## 4. Conclusions

The conducted research allows the following conclusions to be reached:

The density of composites changed with filler content and the type of wood. A reduction of approximately 3–5% in density was observed for the 10% fill composites and for 20% fill and an increase of up to 4%. This is mainly due to the structure of wood waste and the density of fillers. However, on the basis of statistical analysis, it was found that the examined variables are of little importance for the tested characteristic.

The introduction of wood filler into composites increased the hardness, which is not directly proportional to the hardness of trees. The highest hardness was characteristic for the composite with 10 and 20% spruce filler (density 700–850 kg/m^3^), which may be related to the particle size of 998.13 μm and the resin presence probably additionally hardening the composite. At the same time, the silicone-spruce composite shows the smallest abrasive wear by an average of approximately 30% compared to deciduous trees fillers. Both the size and type of the tree from which the waste was obtained significantly influences the hardness and abrasion.

For 20% at the higher the resilience and the lower the strain and stress at break was observed. All composites with 20 wt % filling, showed higher resilience and lower values of the characteristics determined in the static tensile test. For the 10% share, there is no unequivocal dependence of the resilience on the filler content in the composite. It seems that in this case both the type of wood and the property gradient resulting from the casting technology are important. The tested characteristics are strongly dependent on the weight fraction of the filler and the properties of the wood waste.

Conducted aging tests in the sea water environment showed that the area covered with algae increased with the increase of the filler, which was related to the type and structure of wood waste. All composites stimulated the proliferation of normal cells of the human body, demonstrating their lack of toxicity on normal fibroblasts, NHDF cells.

The multicriteria analysis proves that the best test results were obtained for composites filled with spruce waste, both with 10% by weight and with 20% by weight.

Further research will allow for a more complete characterization of WPC as materials for use in protective coatings, insulation systems, or packaging.

## Figures and Tables

**Table 1:** Properties of XIAMETER 4234-T4 silicone [19]. A—Xiameter RTV-4234-T-4 BASE; B—Xiameter T-4 curing agent.

Properties	Unit	Value
Ratio mixing A:B		10:1
Density	(g/cm^3^)	1.1
Viscosity	(mPas)	35,000
Hardness	(ShA)	40
Linear shrinkage	(%)	<0.1
Tensile strength	(MPa)	6.7
Tensile strain	(%)	400

**Table 2:** Properties of wood fillers [20,21,22,23].

Properties	Oak	Beech	Spruce	Hornbeam
After drying density (kg/m^3^)	900–1150	820–1270	700–850	660–1200
Flexural strength (MPa)	74–105	74–210	49–136	58–200
Flexural modulus (GPa)	10–13.5	10–18	7.3–21.4	7–17.7
Compressive strength (MPa)	48	41–99	30–79	54–99
Tensile strength (MPa)	50–180	55–180	21–245	24
Impact strength (J/cm^2^)	1–16	3–19	1–11	8–12
Brinell’s hardness (HBW)	34	34	12	29–36

**Table 3:** The mark of samples.

Name	Base	Filler	Filler Content (%)
XS	XIAMETER 4234 - T4	-	-
10 - O		oak	10
20 - O		oak	20
10 - B		beech	10
20 - B		beech	20
10 - S		spruce	10
20 - S		spruce	20
10 - H		hornbeam	10
20 - H		hornbeam	20

**Table 4:** Chemical composition of substitute sea water [31].

Ingredients	Concentration (g/L)
Sodium chloride (NaCl)	24.53
Magnesium chloride (MgCl_2_)	5.2
Sodium sulfate (Na_2_SO_4_)	4
Calcium chloride (CaCl_2_)	1.16
Potassium chloride (KCl)	0.695
Sodium bicarbonate (NaHCO_3_)	0.201
Potassiumbromide (KBr)	0.101
Boric acid (H_3_BO_3_)	0.027
Strontium chloride (SrCl_2_)	0.025
Sodium fluoride (NaF)	0.003

**Table 5:** Results of microscopic research: wood filler and aged samples.

Wood Filler	10%	20%
oak	* 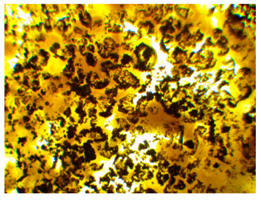 *	* 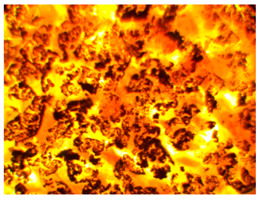 *
beech	* 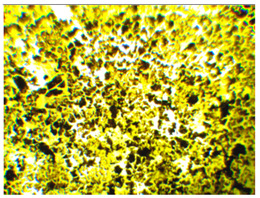 *	* 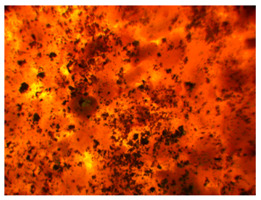 *
spruce	* 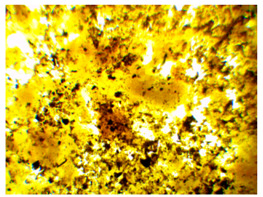 *	* 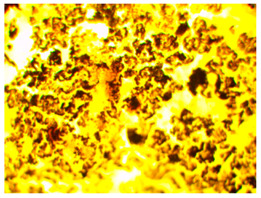 *
hornbeam	* 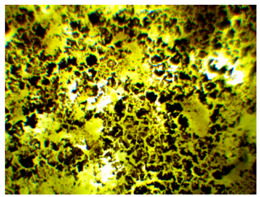 *	* 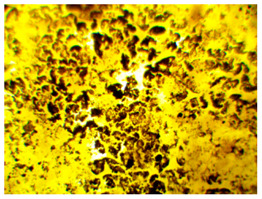 *

**Table 6:** Quality assessment of wood–silicon composites, W_c_—criterion weight, E—evaluation, R—result.

Własność	W_c_	XS				10-O		20-O		10-B		20-B		10-S		20-S		10-H		20-H	
		E		R		E	R	E	R	E	R	E	R	E	R	E	R	E	R	E	R
Density	1	5		5		3	3	4	4	5	5	4	4	2	2	3	3	4	4	5	5
			3		3																
Resilience	4	1		4		2	8	3	12	5	3	5	20	5	20	4	16	4	16	3	12
Abrasion resistance results	6	1		6		4	24	3	18	3	18	2	12	5	30	5	30	4	24	2	12
Hardness	3	1		3		2	6	2	6	4	12	3	9	5	15	5	15	3	9	4	12
Strain at break	2	5		10		3	6	3	6	4	8	2	4	2	4	2	4	1	2	4	8
Stress at break	5	5		25		3	15	4	20	4	20	3	15	1	5	1	5	2	10	2	10
Suma				53			62		66		66		64		76		73		65		59
					51																
